# Diagnostic model development for schizophrenia based on peripheral blood mononuclear cell subtype-specific expression of metabolic markers

**DOI:** 10.1038/s41398-022-02229-w

**Published:** 2022-10-30

**Authors:** Jihan K. Zaki, Santiago G. Lago, Nitin Rustogi, Shiral S. Gangadin, Jiri Benacek, Geertje F. van Rees, Frieder Haenisch, Jantine A. Broek, Paula Suarez-Pinilla, Tillmann Ruland, Bonnie Auyeung, Olya Mikova, Nikolett Kabacs, Volker Arolt, Simon Baron-Cohen, Benedicto Crespo-Facorro, Hemmo A. Drexhage, Lot D. de Witte, René S. Kahn, Iris E. Sommer, Sabine Bahn, Jakub Tomasik

**Affiliations:** 1 Department of Chemical Engineering and Biotechnology, University of Cambridge, Cambridge, UK; 2 Department of Biomedical Sciences of Cells & Systems, University Medical Center Groningen (UMCG), University of Groningen, Groningen, The Netherlands; 3 Department of Psychiatry, Marqués de Valdecilla University Hospital, IDIVAL, School of Medicine, University of Cantabria, Santander, Spain; 4 Centro de Investigación Biomédica en Red de Salud Mental (CIBERSAM), Santander, Spain; 5 University Hospital Münster, Münster, Germany; 6 Autism Research Centre, Department of Psychiatry, University of Cambridge, Cambridge, United Kingdom; 7 Foundation Biological Psychiatry, Sofia, Bulgaria; 8 Cambridgeshire and Peterborough NHS Foundation Trust, Cambridge, United Kingdom; 9 Department of Psychiatry, School of Medicine, University Hospital Virgen del Rocio, IBiS, Sevilla, Spain; 10 Centro de Investigación Biomédica en Red de Salud Mental (CIBERSAM), Sevilla, Spain; 11 Department of Immunology, Erasmus Medical Center, Rotterdam, The Netherlands; 12 Department of Psychiatry, Icahn School of Medicine at Mount Sinai, New York, NY USA; 13 Department of Psychiatry, University Medical Center Utrecht, Utrecht, The Netherlands; 14 Department of Psychiatry, University Medical Center Groningen, University of Groningen, Groningen, The Netherlands

**Keywords:** Diagnostic markers, Schizophrenia

## Abstract

A significant proportion of the personal and economic burden of schizophrenia can be attributed to the late diagnosis or misdiagnosis of the disorder. A novel, objective diagnostic approaches could facilitate the early detection and treatment of schizophrenia and improve patient outcomes. In the present study, we aimed to identify robust schizophrenia-specific blood biomarkers, with the goal of developing an accurate diagnostic model. The levels of selected serum and peripheral blood mononuclear cell (PBMC) markers relevant to metabolic and immune function were measured in healthy controls (*n* = 26) and recent-onset schizophrenia patients (*n* = 36) using multiplexed immunoassays and flow cytometry. Analysis of covariance revealed significant upregulation of insulin receptor (IR) and fatty acid translocase (CD36) levels in T helper cells (*F* = 10.75, *P* = 0.002, *Q* = 0.024 and *F* = 21.58, *P* = 2.8 × 10^−5^, *Q* = 0.0004, respectively), as well as downregulation of glucose transporter 1 (GLUT1) expression in monocytes (*F* = 21.46, *P* = 2.9 × 10^−5^, *Q* = 0.0004). The most robust predictors, monocyte GLUT1 and T helper cell CD36, were used to develop a diagnostic model, which showed a leave-one-out cross-validated area under the receiver operating characteristic curve (AUC) of 0.78 (95% CI: 0.66–0.92). The diagnostic model was validated in two independent datasets. The model was able to distinguish first-onset, drug-naïve schizophrenia patients (*n* = 34) from healthy controls (*n* = 39) with an AUC of 0.75 (95% CI: 0.64–0.86), and also differentiated schizophrenia patients (*n* = 22) from patients with other neuropsychiatric conditions, including bipolar disorder, major depressive disorder and autism spectrum disorder (*n* = 68), with an AUC of 0.83 (95% CI: 0.75–0.92). These findings indicate that PBMC-derived biomarkers have the potential to support an accurate and objective differential diagnosis of schizophrenia.

## Introduction

Schizophrenia is a severe mental health condition, which currently affects approximately 20 million people around the world [1]. It is consistently ranked amongst the most debilitating disorders [2] and is estimated to account for 13.4 million years of life lived with a disability annually [1]. The severity of the disease, as well as its debilitating symptoms, cause a significant burden not only to the affected individuals but also to society at large, despite the relatively low prevalence. The annual direct and indirect costs of schizophrenia in the UK alone are estimated at 6.7 billion pounds, with the cost of lost productivity due to unemployment, absence from work, and premature mortality of patients amounting to 3.4 billion pounds [3]. With the prevalence of schizophrenia continuously rising [1], both the personal and economic costs are expected to increase accordingly. These negative impacts of the disease could be averted with earlier and more accurate diagnostic tools for schizophrenia, as its early diagnosis is associated with improved long-term outcomes [4].

One of the greatest challenges to the effective treatment and diagnosis of schizophrenia is the lack of understanding of the biological mechanisms of the disease. Schizophrenia is considered a highly heterogenous disorder [5], and is often misdiagnosed with other mental health conditions [6, 7]. For instance, it is estimated that 24% of schizophrenia patients are initially misdiagnosed, primarily with bipolar disorder [6, 8]. The current diagnostic procedures involve interview-based methods in accordance with guidelines such as the Diagnostic and Statistical Manual of Mental Disorders (DSM), 5th edition [9, 10], and the International Statistical Classification of Diseases and Related Health Problems (ICD), 11th revision [10, 11]. Therefore, diagnosis is often impacted by subjective interpretation of symptoms, and may be inconsistent between psychiatrist evaluations. For example, the interrater agreement when diagnosing schizophrenia according to ICD-10 and DSM-IV guidelines is at best moderate, with Cohen’s kappa coefficients ranging from 0.56 to 0.59 on a 0–1 scale [12]. In addition, psychotic symptoms of schizophrenia usually appear after a pre-psychotic prodromal stage, when initial symptoms of schizophrenia may be indistinguishable from those of certain affective disorders [13], further complicating early diagnosis. Moreover, while there are currently no preventive measures protecting from developing schizophrenia, evidence suggests that responses to existing treatments in first-episode patients are superior to those of chronic patients, and that biological effects are strongest following early interventions [14, 15]. Considering that current estimates for the mean duration of untreated psychosis range from 22 to over 150 weeks [16], these factors highlight the pressing need for novel, objective diagnostics capable of differential diagnosis at the early stages of schizophrenia.

To this end, the discovery of an objective diagnostic procedure based on biological phenomena could significantly improve the diagnostic milieu of schizophrenia. Biomarker-based technologies could provide novel, inexpensive, and efficient diagnostic tools which overcome the limitations of traditional psychiatrist evaluations, such as the limited availability of psychiatrists, high costs of individual evaluations, and subjective assessments of patients, which could benefit both patients and healthcare systems. This could be implemented either in the form of supporting evidence for psychiatrist evaluation as part of a clinical triage process, or as an objective standalone diagnostic method, depending on the accuracy and economic expense of the procedure. Additionally, the discovery of biomarkers of schizophrenia could advance the biological understanding of disease pathways, and indicate new targets for therapeutic or prognostic developments.

In the present work, we aimed to develop a diagnostic model for schizophrenia based on circulating blood biomarkers selected due to their past association with the disease, to facilitate early diagnosis. To this end, we used multiplexed immunoassay and flow cytometry analyses of schizophrenia-, immune system- and metabolic function-related analytes in serum and peripheral blood mononuclear cells from patients with recent-onset schizophrenia and unaffected healthy individuals. Analysis of covariance was used to identify biomarkers of the disease, and logistic regression was used to build the diagnostic model, which was further evaluated in independent schizophrenia and other relevant neuropsychiatric patient cohorts.

## Methods

### Study population

Samples and data analysed in the present study were collected as part of the clinical trial of simvastatin supplementation in schizophrenia (ClinicalTrials.gov identifier: NCT01999309 [17, 18]). The study population consisted of 119 participants between 18 and 50 years old diagnosed with schizophrenia spectrum disorder using the Diagnostic and Statistical Manual of Mental Disorders, fourth edition (DSM-IV) [19] guidelines. Only patients with recent-onset psychosis were enroled, and therefore the maximum duration of psychosis at the start of the trial was 3 years. The trial was conducted at the University Medical Center Utrecht (UMCU) and University Medical Center Groningen (UMCG) in the Netherlands and participants were recruited between November 2013 and February 2019. The study was approved by the research and ethics committee of the UMCU, protocol number 13–249. Healthy control samples were collected as part of the “Investigating normal variability in brain volumes and cognition in a healthy population” study (ABR NL50657.041.14), carried out at UMCU in parallel to the clinical trial of simvastatin, and using the same recruitment and sample preparation procedures. Informed consent was obtained from all study participants. In the current study, only data from participants who consented to international material and data transfer were analysed, which equated to 26 healthy control individuals and 36 schizophrenia patients. Further details of the trial, including the exact inclusion and exclusion criteria, can be found in previously published reports [17, 18].

### Sample collection and preparation

Collected specimens included serum and peripheral blood mononuclear cell (PBMC) samples. Sample preparation was carried out by the biobank at the UMCU in accordance with the standard operating procedures. Serum samples were collected into 9 ml serum separator tubes. The blood samples were allowed to clot for 60 min, followed by centrifugation at 2000×*g* for 10 min. After that, the supernatants were aliquoted into 0.5 ml tubes and stored at −80 °C until analysis.

Blood samples for PBMC analysis were collected into 9 ml sodium heparin tubes, and PBMC isolation was completed within 24 h of sample collection. The anticoagulated blood samples were diluted with phosphate-buffered saline (PBS) solution at a 1:2 ratio and transferred onto Ficoll-Paque. Next, the samples were centrifuged at 1000 × *g* for 20 min at room temperature. The resulting plasma layer was removed to prevent contamination with platelets, and the PBMC layer was extracted. PBMCs were washed twice with 10 ml of PBS at 250 × *g* for 10 min and resuspended in 1 ml of Roswell Park Memorial Institute (RPMI)-1640 containing 1% penicillin-streptomycin. Samples were cryopreserved in liquid nitrogen following the addition of dimethyl sulfoxide (DMSO) as a cryoprotectant at a 10% concentration.

### Laboratory procedures and analyte measurements

#### Multiplexed immunoassays

Analytes evaluated in the current study were selected based on previous association to schizophrenia [20–22] or metabolic syndrome [23, 24]. Serum analyte measurements were conducted using the Luminex MAGPIX multiplexed immunoassay platform (Luminex Corporation). Target analytes included apolipoproteins (Apo) A1, A2, B, C2 and E (ProcartaPlex Human Apolipoprotein Panel 5-plex, Thermo Fisher, EPX050–15818–901), interleukin 6 (IL-6) and tumour necrosis factor-alpha (TNF-a) (Human Magnetic Luminex Performance Assay, High Sensitivity Cytokine A, R&D Systems, LHSCM000), and serpin E1, chemokine ligand 2 (CCL-2), leptin, adiponectin, C-reactive protein (CRP), resistin and complement factor D (Human Obesity Luminex Performance Assay, R&D Systems, LOBM000). Sample analysis was conducted in accordance with the manufacturers’ instructions. Optimised dilutions were used for sample assaying, and analyte concentrations were calculated using five-point logistic standard curves run in duplicates. Sample measurements were carried out in duplicates in a 96-well plate format and each plate contained three quality control samples. Experimenters were blinded to sample diagnostic allocation.

#### Flow cytometry

PBMCs were thawed at 37 °C, washed with complete Roswell Park Memorial Institute (RPMI) medium (RPMI-1640 with sodium bicarbonate (Sigma-Aldrich), 10% foetal bovine serum (Life Technologies), 50 U/ml penicillin and 50 µg/ml streptomycin (Life Technologies), and 2 mM l-alanyl-l-glutamine dipeptide (Life Technologies)) with 20 µg/ml deoxyribonuclease (Sigma-Aldrich), and resuspended at 1 × 10^6^ cells/ml. Cells were plated at 0.2 ml/well in 96-well plates (Starlab), and stored at 4 °C until analysis on the same day. For staining, PBMCs were washed and resuspended in FACS buffer (PBS with 0.5% bovine serum albumin (Sigma-Aldrich)) with 20% human Fc receptor binding inhibitor (eBioscience). The cells were then incubated for 20 min at room temperature to allow non-specific antibody binding. Next, PBMCs were stained in a total volume of 90 µl using 0.5 µl of anti-human CD3 (UCHT1) PE-Cy7 (eBioscience), 0.5 µl of anti-human CD4 (SK3) PerCP-eFluor 710 (eBioscience), 0.5 µl of anti-human CD8 (SK1) APC-eFluor 780 (eBioscience) and 0.3 µl of anti-human CD14 (MφP9) V500 (BD Biosciences). Additionally, for metabolic marker-stained samples, 10 µl of anti-human glucose transporter 1 (GLUT1, clone 202915) FITC (R&D Systems), 20 µl of anti-human insulin receptor (IR, or CD220, clone 3B6/IR) PE (BD Biosciences) and 2.5 µl of anti-human fatty acid translocase (CD36, clone NL07) eFluor660 (Thermo Fisher) were added as per manufacturer instructions. Unstained control sample wells were filled with equivalent volumes of FACS buffer. Staining was conducted in the dark for 45 min at room temperature. Lastly, cells were washed twice using 200 µl of FACS buffer, resuspended in 150 µl of FACS buffer with 1 µM DAPI (Sigma-Aldrich), and stored at 4 °C until acquisition.

PBMCs were acquired using FACSVerse flow cytometer (BD Biosciences) with 405, 488 and 640 nm laser excitations at a mean flow rate of 2 µl/s. Each PBMC sample was measured once. Photomultiplier tube detector voltage standardisation and quality control throughout multiple experimental runs were conducted using Multicolour Cytometer Setup and Tracking beads (BD Biosciences). Anti-mouse IgGκ antibody capture beads (Bangs Laboratories) labelled separately with anti-human CD3 (UCHT1) PE-Cy7, anti-human CD4 (SK3) PerCP-eFluor 710, anti-human CD8 (SK1) APC-eFluor 780, anti-human CD14 (MφP9) V500, anti-human GLUT1 (202915) FITC, anti-human CD220 (IR) (3B6/IR) PE and anti-human CD36 (NL07) eFluor660, together with single stain controls stained with DAPI, were used for fluorescence compensation. Experimenters were blinded to sample diagnostic allocation.

### Data analysis

#### Clinical data processing

Data processing and statistical analysis was performed using R v.4.0.5. Clinical and demographic characteristics of all used datasets were compared between the patient and control groups using the R ‘tableone’ package. Statistically significant differences between the groups were determined using the Mann–Whitney *U*-test for continuous variables, and Fisher’s exact test for categorical variables (Pearson’s chi-squared test in comparisons with more than two groups). The significance threshold was set to *P* < 0.05, and all tests were two-tailed.

#### Serum data processing

Raw immunoassay data processing was conducted using xPONENT software 4.1 (Luminex Corporation), followed by analysis in R. Serum analytes with more than 30% of values outside the linear range of the assays, as well as samples with a coefficient of variation (CV) between replicate measurements above 50%, were excluded from the analysis. Among the included analytes, the highest proportion of missing values was observed for IL-6 (7%), and the average CV across replicate samples (±standard deviation) was 6.6 ± 11.7%. For analytes remaining in the dataset, values outside the linear assay range (1.6% of all measured values) were replaced with the concentrations at the respective standard limits, i.e. the lower and upper limits of quantitation. Between-plate batch effects caused by performing the experiments over multiple days and across multiple plates were evaluated using the Kruskal–Wallis test. Within-plate effects were assessed using Spearman’s rank correlation coefficient against sample acquisition order. The respective batch effects were removed by applying Z-factor scaling across plates, and linear regression within plates.

#### PBMC data processing

The raw flow cytometry data were analysed using FlowJo v.10.8 software (Tree Star) and exported to R. PBMC samples with a viability of 60% or less, or a live cell count below 100 per population, were excluded from the analysis. For the remaining data, stain indexes were calculated separately for the schizophrenia and control groups as the ratio of the mean MFI (median fluorescence intensity) of the antibody-stained sample and the mean MFI of the unstained sample, for each cell subtype and marker separately. Only metabolic markers that in both groups showed staining of more than 10% above the unstained control fluorescence were analysed.

#### Patient vs. control analysis

Analyte association with schizophrenia status was evaluated using analysis of covariance (ANCOVA), adjusting for covariates, which differed significantly between the schizophrenia and control groups, as well as for batch effects and cell counts in PBMC metabolic marker analysis. Analysis of variance (ANOVA) was used to determine unadjusted estimates. *P* values were corrected for multiple comparisons (shown as *Q* values) using permutation testing (*n* = 10,000 permutations). The significance threshold was set to *Q* < 0.05, and all tests were two-tailed. Analyte association to covariates that differed significantly between the groups in any of the cohorts was evaluated using the Mann–Whitney *U*-test, the Kruskal–Wallis test, and linear regression.

#### Diagnostic model development

Analytes significantly associated with schizophrenia in the ANCOVA analysis were used to create a multivariable diagnostic model. The model was developed using logistic regression and leave-one-out cross-validation. Model performance was evaluated using two additional, non-overlapping datasets. The first validation set contained PBMC biomarker data collected from first-onset antipsychotic drug-naïve schizophrenia patients (*n* = 34) and matched unaffected controls (*n* = 39) from the University Hospital Marqués de Valdecilla, Santander, Spain [25]. The dataset was used to evaluate and tune the original model. The second validation set referred to as the 'psychiatric spectrum dataset', contained PBMC data from previously treated donors with three different mental health conditions, including autism spectrum condition (*n* = 25; Cambridge Autism Research Centre, Cambridge University, Cambridge, UK), bipolar disorder (*n* = 25; Foundation Biological Psychiatry, Sofia, Bulgaria, and Union House, Cambridgeshire and Peterborough Mental Health Foundation Trust, Cambridge, UK), and major depressive disorder (*n* = 25; Westfälische Wilhelms University Hospital, Münster, Germany), as well as drug-naïve patients with schizophrenia (*n* = 25; University Hospital Marqués de Valdecilla, Santander, Spain) [22, 26]. Additionally, the psychiatric spectrum dataset included 100 matched healthy controls, i.e. 25 for each condition. The psychiatric spectrum dataset was used to evaluate the performance of the final model and its specificity to schizophrenia. The medical faculty ethical committees responsible for the respective sample collection sites in the validation cohorts approved the study protocols. Written informed consent was given by all participants. Biomarker data were standardised across the three cohorts using Z-factor scaling for fitting and testing the model. Following data processing and filtering as described above, 24 autism spectrum condition, 21 bipolar disorder, 23 major depressive disorder, 22 schizophrenia and 84 control samples were included in the analysis. Model performance was evaluated using the area under the receiver operating characteristic curve (AUC), and an optimal classification cut-off was determined using Youden’s J statistic.

## Results

### Demographic and clinical data comparison

The demographic and clinical characteristics of the study participants from the original cohort are presented in Table 1, and for participants from the validation sets in Supplementary Tables 1, 2. The comparison revealed that age, education, exposure to childhood abuse, smoking and cognition differed significantly between the schizophrenia and control groups in the original cohort. Compared to the control group, schizophrenia patients were older, had lower education levels, higher exposure to childhood abuse, a higher proportion of smokers, and lower cognitive scores measured using the Brief Assessment of Cognition in Schizophrenia (BACS) scale. Within the validation datasets, patients with schizophrenia from the first validation cohort had a significantly lower body mass index (BMI) than healthy controls, while in the second, psychiatric spectrum dataset, they were significantly younger and had a lower BMI compared to patients with major depressive disorder. Variables that differed significantly between the groups were controlled for in the subsequent analysis, except for the total BACS score, which reflects schizophrenia symptoms.

**Table 1:** Demographic and clinical characteristics of healthy control participants and schizophrenia patients.

Characteristic	Control	Schizophrenia	*P* value	Missing
	*n* = 26	*n* = 36		(%)
Sex, No. (%)				
Female	4 (15)	8 (22)	0.729	0
Male	22 (85)	28 (78)		
Age, median years, [IQR]	22.0 [21.0,24.8]	26.0 [22.0,30.2]	0.021*	0
Highest level of education, No. (%)^a^				
Primary	0 (0)	2 (6)	<0.001***	3.2
Secondary	2 (8)	15 (42)		
College	13 (54)	19 (53)		
University	9 (38)	0 (0)		
Nationality, No. (%)				
Dutch Caribbean	2 (8)	0 (0)	0.156	3.2
Iran	0 (0)	1 (3)		
Netherlands	22 (92)	35 (97)		
Duration of illness, years, median [IQR]		1.0 [0.8,2.0]		0
Childhood abuse, No. (%)^b^	1 (4)	14 (39)	0.005**	1.6
Rating scales, median, [IQR]				
PANSS positive		13.0 [9.0,17.2]		0
PANSS negative		14.0 [10.8,19.0]		0
PANSS general		29.0 [26.0,33.0]		0
PANSS total		59.0 [46.8,67.5]		0
GAF		55.0 [50.0,63.5]		1.6
CDSS		3.0 [0.0,4.5]		1.6
BACS total^c^	0.4 [0.2, 0.7]	−0.2 [−0.5, 0.1]	<0.001***	0
Medication, No. (%)^d^				
Amisulpride		2 (6)		0
Aripiprazole		9 (25)		0
Clozapine		4 (11)		0
Haloperidol		3 (8)		0
Lorazepam		3 (8)		0
Methylphenidate		2 (6)		0
Olanzapine		8 (22)		0
Paliperidone		4 (11)		0
Quetiapine		5 (14)		0
Alcohol consumption, No. (%)	21 (88)	27 (75)	0.392	3.2
Portions/week, median [IQR]	4.0 [1.5,6.0]	2.0 [0.0,4.2]	0.04*	4.8
Recreational drug use, No. (%)	5 (21)	9 (25)	0.95	3.2
Smoking, No. (%)	4 (17)	24 (67)	0.001**	4.8
Cigarettes/week, median [IQR]	0.0 [0.0,0.0]	8.0 [0.0,14.2]	<0.001***	4.8
Coffee consumption, No. (%)	17 (71)	28 (78)	0.761	3.2
Cups/week, median [IQR]	2.0 [1.0,3.0]	2.0 [1.0,4.0]	0.883	6.5
Clinical characteristics, median [IQR]				
Systolic blood pressure, mmHg	122.0 [115.0,135.0]	122.0 [115.8,133.5]	0.587	1.6
Diastolic blood pressure, mmHg	75.0 [69.0,81.0]	76.0 [71.0,81.2]	0.528	1.6
Weight, kg	76.0 [74.0,82.0]	74.5 [66.8,89.0]	0.854	1.6
Height, cm	181.0 [175.0,185.0]	179.5 [173.8,184.2]	0.39	1.6
Body mass index, kg/m^2^	23.8 [21.1,25.8]	24.1 [20.6,26.7]	0.66	1.6
Waist circumference, cm	85.0 [82.0,88.0]	90.0 [84.0,99.5]	0.099	3.2
Metabolic syndrome, No. (%)^e^	2 (8)	2 (6)	1	0

*IQR* interquartile range, *PANSS* positive and negative syndrome scale in schizophrenia, *GAF* global assessment of functioning scale, *CDSS* Calgary depression scale for schizophrenia, *BACS* brief assessment of cognition in schizophrenia scale.

*P < 0.05, **P < 0.01, ***P < 0.001.

^a^‘College’ includes the Dutch middelbaar beroepsonderwijs (MBO) and hoger beroepsonderwijs (HBO) education; ‘University’ refers to the Dutch wetenschappelijk onderwijs (WO) education.

^b^Determined based on the Childhood Trauma Questionnaire question 'Do you believe that you have been physically, emotionally, or sexually abused?'

^c^Calculated as the mean of scaled BACS component scores.

^d^Only medications used by more than one participant are shown.

^e^Determined according to Jin and Benyshek (2013) [50].

### Biomarkers of schizophrenia

**Fig. 1:**
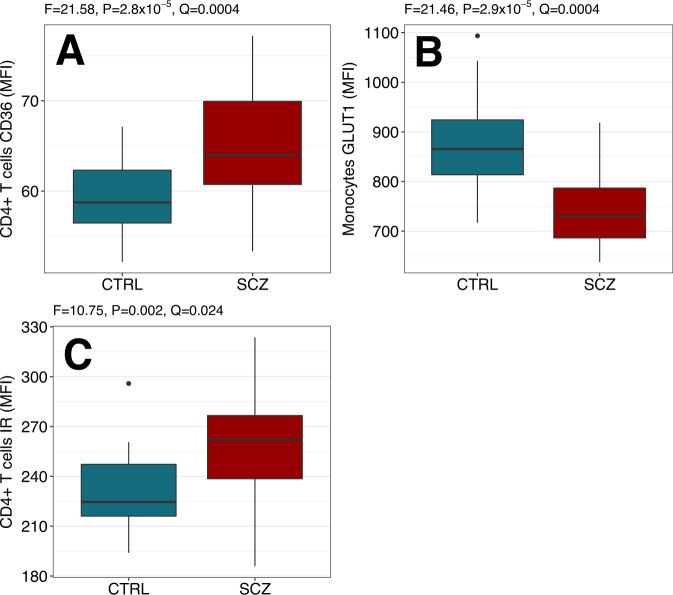
Biomarkers of schizophrenia. Shown are significant (*Q* < 0.05) results from the ANCOVA analysis of blood biomarker data in the recent-onset schizophrenia (SCZ, red) and healthy control (CTRL, cyan) groups from the original cohort. Y-axes show median fluorescence intensity (MFI) of metabolic marker staining in PBMC subtypes, including fatty acid translocase (CD36) expression in T helper cells (**A**), glucose transporter 1 (GLUT1) expression in monocytes (**B**), and insulin receptor (IR) expression in T helper cells (**C**). Boxplots represent the median, interquartile range, and minimum and maximum values, excluding outliers (dots). The analysis was adjusted for batch effects, cell counts, age, smoking, education and childhood abuse.

Among all measurements, levels of three PBMC epitopes were found to be significantly altered in schizophrenia after adjusting for covariates and accounting for multiple comparisons. The significant findings included increased CD36 expression in T helper cells (*F* = 21.58, *P* = 2.8 × 10^−5^, *Q* = 0.0004), decreased GLUT1 expression in monocytes (*F* = 21.46, *P* = 2.9 × 10^−5^, *Q* = 0.0004), and higher expression of IR in T helper cells (*F* = 10.75, *P* = 2.0 × 10^−3^, *Q* = 0.024). Additionally, ANOVA analysis showed that the Q values remained significant for both monocyte GLUT1 (*Q* = 0.0017) and T helper cell CD36 (*Q* = 0.036) analytes when unadjusted for covariates, while IR in T helper cells was borderline significant (*Q* = 0.068). The PBMC expression of significant biomarkers across the groups is shown in Fig. 1. No other significant differences were observed between the schizophrenia and control groups in serum or PBMC analyte levels.

### Diagnostic model build

**Fig. 2:**
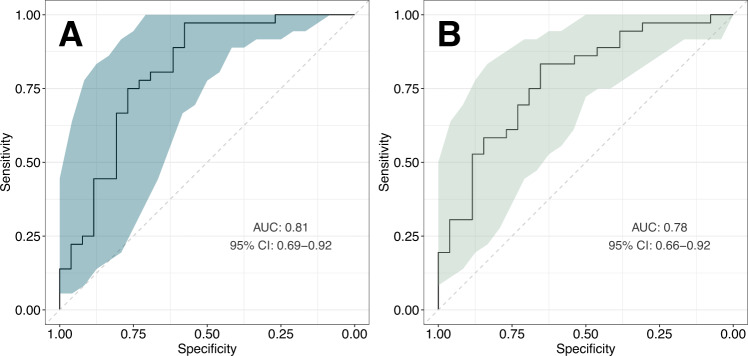
Performance of the biomarker-based multivariable diagnostic model in separating recent-onset schizophrenia patients (*n* = 36) from healthy controls (*n* = 26) in the original cohort. Shown are receiver operating characteristic (ROC) curves of the three-biomarker model (**A**) and two-biomarker model (**B**), with sensitivity confidence intervals (CI) marked in teal and light green, respectively. The area under the ROC curve (AUC) was calculated using leave-one-out cross-validation.

A multivariable diagnostic model was built using the three significant biomarkers of schizophrenia identified in the patient vs. control comparison, i.e., monocyte GLUT1 and T helper cell IR and CD36, adjusted for cell counts. The trained logistic regression algorithm showed a cross-validated AUC of 0.81 (95% CI: 0.69–0.92) in separating schizophrenia patients from healthy control individuals in the original dataset. While monocyte GLUT1 and T helper cell CD36 were the strongest predictors, consistent with having the lowest *P* values in the biomarker discovery phase, T helper cell IR had a minimal effect on the model performance and its removal resulted in a cross-validated AUC of 0.78 (95% CI: 0.66–0.92), similar to the original model. Therefore, two versions of the model were further tested, one including all three predictors, and one with only the two strongest predictors, i.e. monocyte GLUT1 and T helper cell CD36. The receiver operating characteristic curve (ROC) for both models are presented in Fig. 2.

### Model optimisation

**Fig. 3:**
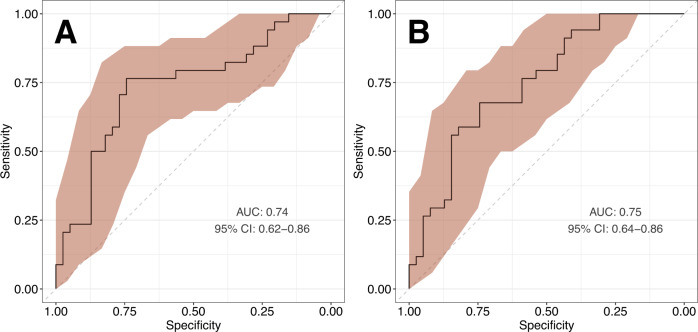
Validation of the diagnostic model in an independent cohort of 34 first-onset drug-naïve schizophrenia patients and 39 healthy controls. Shown are receiver operating characteristic curves (ROC) of the three-biomarker model (**A**) and two-biomarker model (**B**), with sensitivity confidence intervals (CI) marked in red. AUC area under the ROC curve.

The three- and two-biomarker models were next tested in an independent validation cohort of 34 first-onset drug-naïve schizophrenia patients and 39 matched healthy controls. The three-biomarker model showed an AUC of 0.74 (95% CI: 0.62–0.86, and the two-biomarker model showed an AUC of 0.75 (95% CI: 0.64–0.86) when separating the schizophrenia and control groups. Model performance in the validation dataset is presented in Fig. 3. To avoid overfitting of the model and due to better performance in the validation dataset, the two-biomarker model was selected as the optimal model for further testing.

### Model validation

**Fig. 4:**
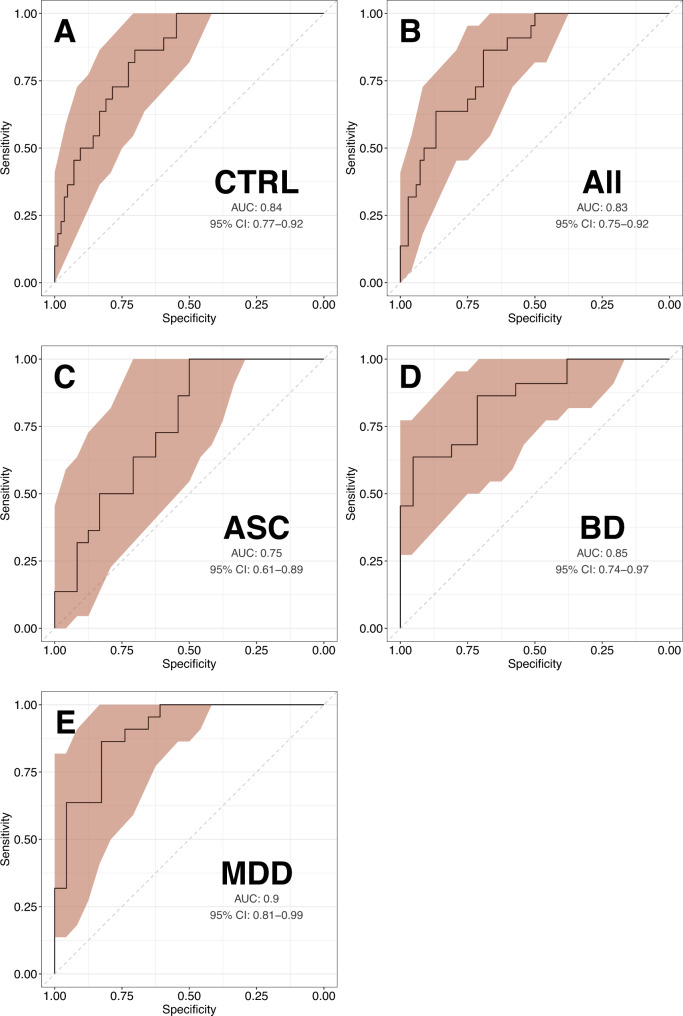
Model performance in the psychiatric spectrum validation dataset. Plots show receiver operating characteristic curves (ROC) from applying the final, two-biomarker model to differentiate first-onset drug-naïve schizophrenia patients (*n* = 22) from healthy control individuals (CTRL, *n* = 84; **A**) as well as from patients with three other major neuropsychiatric conditions combined (All, *n* = 68; **B**), including autism spectrum condition (ASC, *n* = 24; **C**), bipolar disorder (BD, *n* = 21; **D**) and major depressive disorder (MDD, *n* = 23; **E**). Sensitivity confidence (CI) intervals are marked in red. AUC area under the ROC curve.

To evaluate the final, two-biomarker model in a more naturalistic clinical scenario, the model was tested in an independent psychiatric spectrum dataset consisting of patients with four major mental health conditions and a healthy control group. The model showed an AUC of 0.84 (95% CI: 0.77–0.92) when separating first-onset drug-naïve schizophrenia patients (*n* = 22) from healthy control individuals (*n* = 84), 0.83 (95% CI: 0.75–0.92) when separating participants with schizophrenia from those with other major psychiatric conditions combined (*n* = 68), 0.75 (95% CI: 0.61–0.89) when distinguishing schizophrenia from autism spectrum condition (*n* = 24), 0.85 (95% CI: 0.74–0.97) when distinguishing schizophrenia from bipolar disorder (*n* = 21), and 0.90 (95% CI: 0.81–0.99) when distinguishing schizophrenia from major depressive disorder (*n* = 23). Model performance in the psychiatric spectrum validation dataset is presented in Fig. 4. Additional analyses showed that no significant association was observed between the identified candidate biomarkers and the covariates that differed significantly between the clinical groups, as shown in Supplementary Figs. 1–5.

## Discussion

The current study aimed to identify circulating blood biomarkers of schizophrenia in order to develop a robust biomarker-based model that could aid an early and accurate diagnosis of the disease. To this end, serum and PBMC samples were collected from recent-onset schizophrenia patients and healthy controls, and the levels of selected disease-related analytes were compared between the groups. Expression of three PBMC epitopes, monocyte GLUT1 and T helper cell CD36 and IR, was found to be significantly altered in schizophrenia. A diagnostic model based on the two most robust biomarkers, monocyte GLUT1 and T helper cell CD36, demonstrated good predictive performance when separating schizophrenia patients from healthy controls in three independent cohorts, as well as from patients with other relevant neuropsychiatric disorders, including autism spectrum condition, bipolar disorder and major depressive disorder.

Downregulation of GLUT1 in monocytes may indicate impaired energy metabolism in schizophrenia. GLUT1 is a protein responsible for glucose transport across cell membranes. Unlike other GLUT proteins, GLUT1 is regulated by circulating blood glucose rather than insulin [27]. Since glucose starvation is known to upregulate GLUT1, lower GLUT1 expression in monocytes may be linked to hyperglycaemia and other glucoregulatory abnormalities which are frequently observed in schizophrenia patients [22, 28]. Features such as insulin resistance have been reported in both treated and drug-naïve patients and linked to schizophrenia genetic risk [25, 29, 30]. Likewise, increased expression of CD36 in T helper cells in schizophrenia may be linked to metabolic abnormalities associated with the disease. CD36 is a transmembrane glycoprotein expressed in multiple blood cell types, which acts as a class B scavenger receptor. Its roles vary between different cell types and include the internalisation of dying human and pathogenic cells, and the facilitation of fatty acid transport into cells [31]. CD36 alterations have been implicated in numerous disorders, such as obesity and inflammatory conditions [32, 33]. Similar to GLUT1, its increased expression has also been associated with abnormal glucose regulation observed in schizophrenia [22]. Importantly, the relatively low incidence of metabolic abnormalities in the investigated cohort suggests that the findings are associated with schizophrenia as such rather than comorbid metabolic dysfunctions.

While the identified biomarkers were found in peripheral blood samples, they could represent similar changes in the central nervous system. GLUT1 is the primary glucose transporter in the brain, alongside GLUT3. Its decreased expression in plasma membranes has been linked to the lowered capacity for glucose transport into the brain and proposed as a potential causal factor in the development of schizophrenia symptoms such as delusions, hallucinations and cognitive effects [27]. Previous studies have suggested that the glucose signalling cascade in the brain is impacted in schizophrenia, where excess insulin may serve as a compensatory mechanism to maintain glycaemic control [34]. Furthermore, proteins associated with the glycolytic pathway have been linked to schizophrenia in the past [35], which supports the notion that metabolic abnormalities could be inherent to at least a subgroup of schizophrenia patients. Recent studies have also suggested that schizophrenia may be a consequence of bioenergetic pathway abnormalities in the brain, indicating potential novel treatment opportunities [36, 37]. Regarding the role of CD36 in the brain, its functions include regulating myelin and amyloid debris clearance by microglia and reducing neuroinflammation [38]. CD36 dysregulation may therefore be linked to immune abnormalities frequently observed in both peripheral tissues and the central nervous system of schizophrenia patients [39]. Moreover, alterations in CD36 expression have been implicated in numerous brain disorders, such as major depressive disorder and Alzheimer’s disease [32]. While the direct causal link between the brain and peripheral abnormalities in schizophrenia remains to be established, future biomarker expression and brain imaging studies are warranted to elucidate their exact role and implications for schizophrenia diagnosis and treatment.

The current results support and expand the findings reported previously by Lago et al. (2021), where the monocyte GLUT1 expression was also reduced in schizophrenia, while T helper cell CD36 expression was increased [22]. Notably, this is despite major differences in patient characteristics between the two studies. Schizophrenia patients in the current analysis were recent-onset patients treated with antipsychotic medication prior to sample collection, as opposed to the first-onset drug-naïve patient population in the previous study. Therefore, monocyte GLUT1 and T helper cell CD36 constitute potential persistent markers of the disease that are not affected by existing treatments. While the previous report revealed also significant alterations in the levels of two other biomarkers, namely CD4^−^CD8^−^ T cell CD36 and monocyte IR, in the drug-naïve patient cohort, these were not reproduced in the current analysis. Instead, we observed an additional biomarker, T helper cell IR, which was significantly upregulated in schizophrenia. Because this finding was specific to treated patients, and the inclusion of T helper cell IR in the diagnostic model decreased model performance in the drug-naïve validation cohort, this marker is most likely associated with antipsychotic treatment effects rather than schizophrenia itself. This is consistent with the well-known effects that antipsychotic medications have on peripheral insulin signalling pathways [40]. Taken together, these results also highlight the cell-subtype specificity of the identified biomarkers, and indicate monocytes and T helper cells as PBMC subtypes of particular relevance to schizophrenia. Although alterations in PBMC subtype frequencies have been linked with schizophrenia in previous publications [41], the specific markers analysed here have not been extensively evaluated in the past. Therefore, further research is required to evaluate the connection between schizophrenia and GLUT1 and CD36 expression in monocytes and T helper cells.

The diagnostic model developed in the current study showed ‘fair’ (AUC > 0.7) to ‘excellent’ (AUC > 0.9) [42] performance in differentiating patients with schizophrenia from those with other relevant mental health disorders with overlapping symptoms. Differentiation of schizophrenia from major depressive disorder and bipolar disorder was particularly notable, with respective AUCs of 0.90 and 0.85. This is an important finding, given that approximately one-third of patients with bipolar disorder receive an incorrect diagnosis of schizophrenia [43]. Compared to previously published biomarker-based algorithms [44–47], the ability of the current model to differentiate schizophrenia from other mental health disorders is unique and can be considered one of the major strengths of the present work [48]. Given that most previously published diagnostic algorithms using biomarker data to diagnose schizophrenia contained a large number of predictors, ranging from 18 to 51 [45, 46, 49], it is notable that the present model achieves relatively high diagnostic performance using only two biomarkers. While the performance of the model is relatively high, it can potentially be further improved by expanding the biomarker panel to include other biological measurements, introducing additional predictors, such as questionnaire or digital data, and employing more advanced machine-learning algorithms in model development. Importantly, the model was validated in three independent cohorts recruited at multiple different locations, which shows promise in overcoming the challenges of irreproducibility. If such tools are extensively validated in larger independent patient groups, they have the potential to substantially enhance future mental healthcare, for example, by supporting psychiatrist-based diagnosis or as pre-screening tools in the clinical triage process. Such an approach could significantly improve current diagnostic procedures, which often take months or years to reach an accurate diagnosis.

The current results should be interpreted within their limitations. First, the limited sample size of the original, discovery dataset allowed for detecting only strong biological signals and precluded the identification of biomarkers with smaller effect sizes. This also resulted in a relatively high uncertainty around the diagnostic performance estimates, as illustrated by the broad ROC confidence intervals. Second, the number of measured variables was relatively high compared to the number of samples. Consequently, several findings that were nominally significant (i.e. *P* < 0.05), such as serum IL-6 or certain apolipoproteins, did not survive the adjustment for multiple comparisons. Third, the patient population in the original cohort, as well as the non-schizophrenia disease groups in the psychiatric spectrum dataset, were not drug-naïve, introducing the possibility of medication-related effects on biomarker levels. Also, data on certain patient characteristics, such as childhood abuse and cognitive scores, were not available for the validation cohorts. Thus, evaluation of their effects on biomarker levels was not possible. Furthermore, the current inclusion criteria might not have fully represented the general psychiatric patient population. Therefore, further studies are required to assess the generalisability of these results. Lastly, the model utilises live cell measurements, which significantly limits its application as a rapid and economically viable diagnostic test.

In conclusion, we identified circulating blood biomarkers of schizophrenia and utilised them to develop an accurate diagnostic model. The identified biomarkers, T helper cell CD36 and monocyte GLUT1, showed significant predictive capability in both drug-naïve and medicated schizophrenia patients, as well as in a trans-diagnostic psychiatric patient cohort, suggesting their potential clinical relevance. With further development using novel machine-learning methods and expanded analyte measurements, such tests could support an earlier and more accurate diagnosis of schizophrenia, and lower the personal and economic burden of the disease.

## Supplementary information

Supplementary material

## Data Availability

The code is available from the corresponding authors upon reasonable request.
