# Supplementary material for: Diagnostic model development for schizophrenia based on peripheral blood mononuclear cell subtype-specific expression of metabolic markers
Source: Transl Psychiatry. 2022 Oct 30;12:457. doi: 10.1038/s41398-022-02229-w (PMC9618570; doi:10.1038/s41398-022-02229-w)
Supplement: Supplementary file 1 — Supplementary material [file 41398_2022_2229_MOESM1_ESM.docx]

**Diagnostic Model Development for Schizophrenia Based on Peripheral Blood Mononuclear Cell Subtype-specific Expression of Metabolic Markers**

Jihan K Zaki^1^ MPhil, Santiago G Lago^1^ PhD, Nitin Rustogi^1^ MRes, Shiral S Gangadin^2^ MSc, Jiri Benacek^1^ PgDip, Geertje F van Rees^1^ PhD, Frieder Haenisch^1^ PhD, Jantine A Broek^1^ PhD, Paula Suarez-Pinilla^3,4^ MD PhD, Tillmann Ruland^5^ MD PhD, Bonnie Auyeung^6^ PhD, Olya Mikova^7^ MD, Nikolett Kabacs^8^ MD, Volker Arolt^5^ MD PhD, Simon Baron-Cohen^6^ PhD, Benedicto Crespo-Facorro^3,4,9,10^ MD PhD, Hemmo A Drexhage MD PhD^11^, Lot D de Witte^12^ MD PhD, Rene S Kahn^12,13^ MD PhD, Iris E Sommer^2,14^ MD PhD, Sabine Bahn*^1^ MD PhD, Jakub Tomasik*^1^ PhD

* Corresponding authors at: Department of Chemical Engineering and Biotechnology, University of Cambridge, Philippa Fawcett Drive, Cambridge CB3 0AS, UK. E-mail address: sb209@cam.ac.uk (S. Bahn), jt455@cam.ac.uk (J. Tomasik).

^1^Department of Chemical Engineering and Biotechnology, University of Cambridge, Cambridge, UK; ^2^Department of Biomedical Sciences of Cells & Systems, University Medical Center Groningen (UMCG), University of Groningen, Groningen, the Netherlands; ^3^Department of Psychiatry, Marqués de Valdecilla University Hospital, IDIVAL, School of Medicine, University of Cantabria, Santander, Spain; ^4^Centro de Investigación Biomédica en Red de Salud Mental (CIBERSAM), Santander, Spain; ^5^University Hospital Münster, Münster, Germany; ^6^Autism Research Centre, Department of Psychiatry, University of Cambridge, Cambridge, United Kingdom; ^7^Foundation Biological Psychiatry, Sofia, Bulgaria; ^8^Cambridgeshire and Peterborough NHS Foundation Trust, Cambridge, United Kingdom; ^9^Department of Psychiatry, School of Medicine, University Hospital Virgen del Rocio, IBiS, Sevilla, Spain; ^10^Centro de Investigación Biomédica en Red de Salud Mental (CIBERSAM), Sevilla, Spain; ^11^Department of Immunology, Erasmus Medical Center, Rotterdam, the Netherlands; ^12^Department of Psychiatry, Icahn School of Medicine at Mount Sinai, New York, NY; ^13^Department of Psychiatry, University Medical Center Utrecht, Utrecht, the Netherlands; ^14^Department of Psychiatry, University Medical Center Groningen, University of Groningen, Groningen, the Netherlands

**Supplementary Material**

**Supplementary Table 1. Demographic characteristics of healthy controls and schizophrenia patients in the validation dataset.** Presented are median values with interquartile ranges, and P values from the Mann-Whitney U test for continuous variables, or Fisher’s exact test for categorical variables. Abbreviations: CTRL, healthy control; IQR, Interquartile range; SCZ, schizophrenia. * P<0.05, ** P<0.01, *** P<0.001.

| **Characteristic** | **CTRL** | **SCZ** | **P-value** | **Missing** |
| --- | --- | --- | --- | --- |
|  | **n=39** | **n=34** |  | **(%)** |
| Sex, No. (%) |  |  |  |  |
| Female | 15 (38) | 14 (41) | 1 | 0 |
| Male | 24 (62) | 20 (59) |  |  |
| Age, median years, [IQR] | 33.4 [28.7,38.5] | 29.8 [23.3,37.6] | 0.159 | 0 |
| Body mass index, kg/m^2^, median [IQR] | 25.4 [23.2,27.8] | 22.4 [21.0,24.3] | 0.003** | 5.5 |
| Cannabis use | 9 (23) | 12 (35) | 0.373 | 0 |
| Smoking | 16 (41) | 18 (53) | 0.434 | 0 |
| Alcohol consumption | 13 (33) | 18 (53) | 0.146 | 0 |

**Supplementary Table 2. Demographic characteristics of healthy controls and patients in the psychiatric spectrum dataset.** Presented are mean values ± standard deviation, and P values from the Kruskal-Wallis test for age and BMI, chi-squared test for sex, as well as significant pairwise comparisons from post-hoc Dunn’s tests. ^1)^ BMI data was missing for 2 patients in the schizophrenia group. Abbreviations: ASC, autism spectrum condition; BD, bipolar disorder; BMI, body mass index; CTRL, healthy control; MDD, major depressive disorder; SCZ, schizophrenia; SD, standard deviation. * P<0.05, ** P<0.01, *** P<0.001.

| **Characteristic** | **CTRL** | **ASC** | **BD** | **MDD** | **SCZ** | **P value** | **Post-hoc tests (vs. SCZ)** |
| --- | --- | --- | --- | --- | --- | --- | --- |
|  | **n=100** | **n=25** | **n=25** | **n=25** | **n=25** |  |  |
| Age, years, mean ± SD | 33.2 ± 10.2 | 31.5 ± 8.3 | 32.6 ± 9.5 | 40.5 ± 10.0 | 27.3 ± 6.7 | 0.0002*** | MDD>SCZ (P<0.001) |
| Sex, Female\|Male | 51\|49 | 11\|14 | 13\|12 | 13\|12 | 8\|17 | 0.492 |  |
| BMI, kg/m^2^, mean ± SD | 24.0 ± 3.9 | 23.9 ± 4.4 | 24.2 ± 4.3 | 26.6 ± 4.5 | 22.9 ± 6.4^1^ | 0.013* | MDD>SCZ (P<0.01) |
| Treatment, naïve\|free\|treated |  | 0\|18\|7 | 0\|3\|22 | 0\|0\|25 | 25\|0\|0 |  |  |


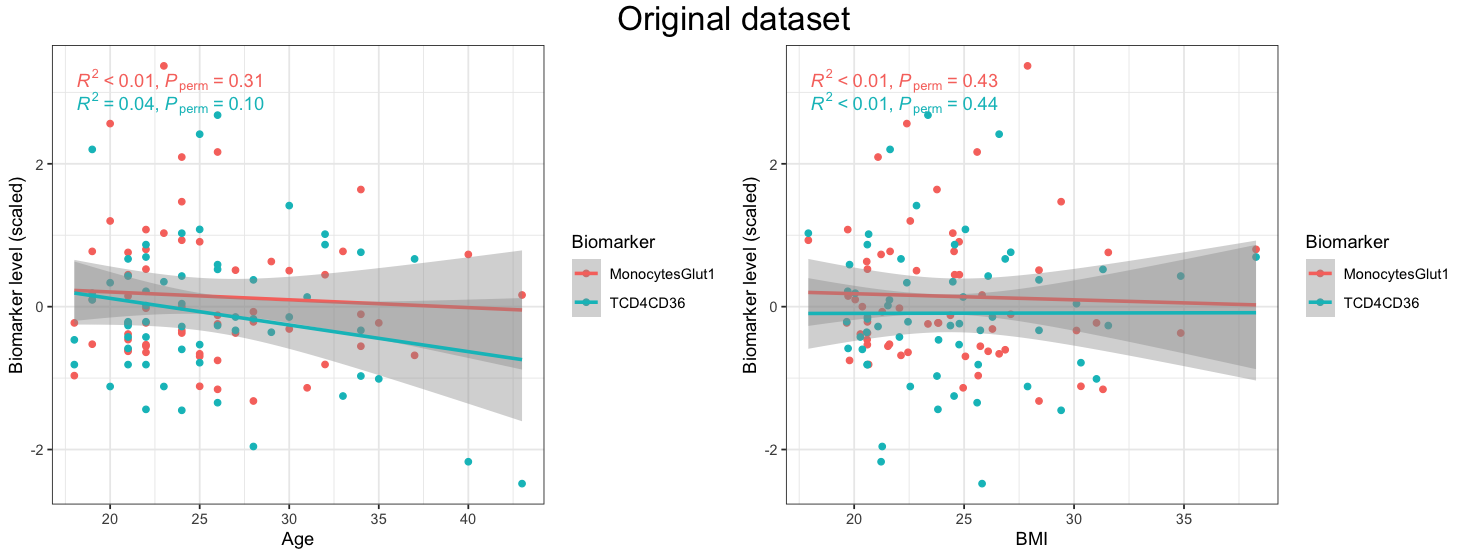

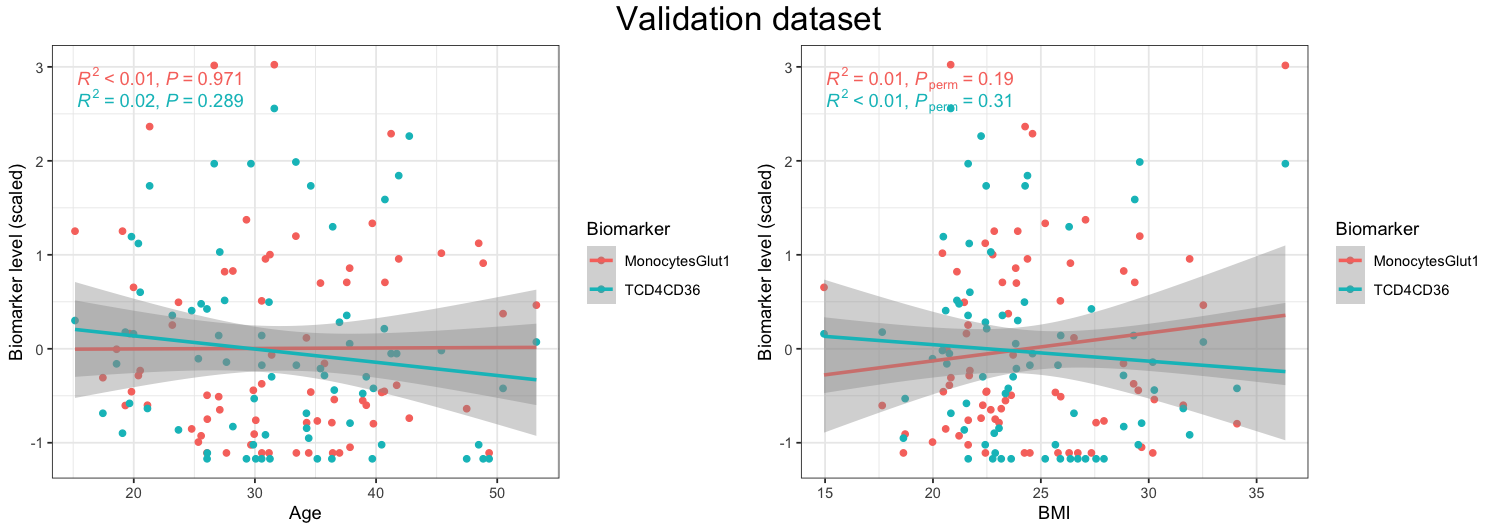


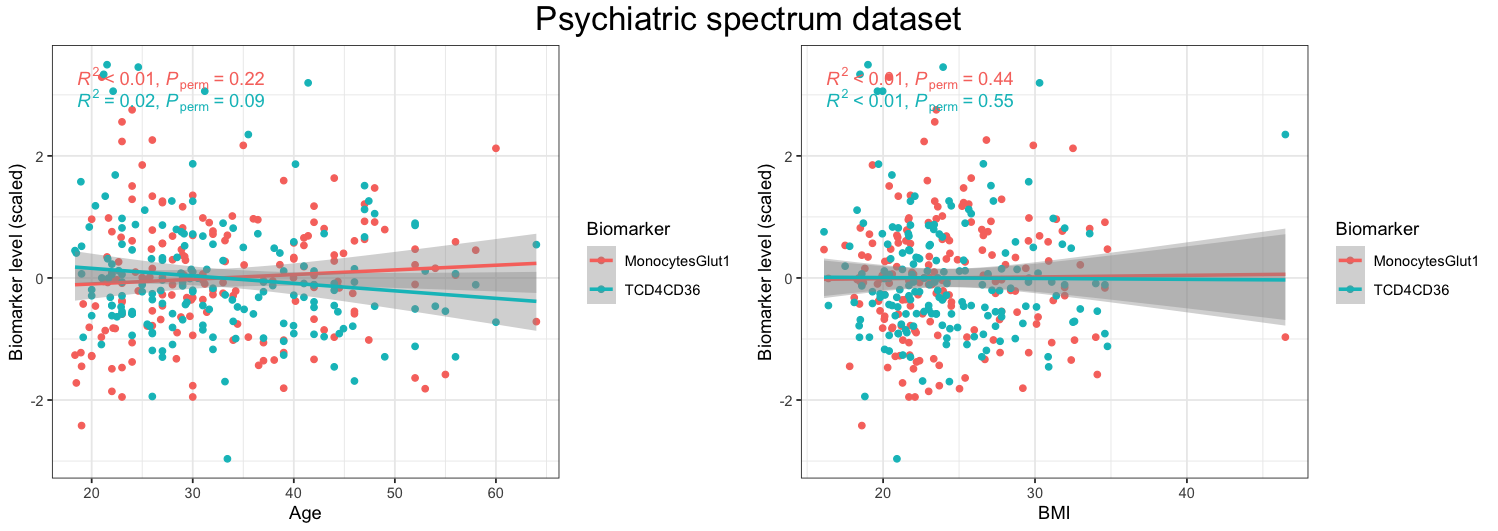


**Supplementary Figure 1. Biomarker levels against age and body mass index** (**BMI) in all three datasets used in the study.** Biomarker levels (Z-scores) are marked on the Y-axes, and age (left) and BMI (right) are shown on the X-axes. Monocyte glucose transporter 1 (GLUT1) is marked in red and CD4^+^ T cell fatty acid translocase (CD36) in blue. A linear regression line is fit for both biomarkers against both covariates, 95% confidence intervals are represented by the shaded areas, and P values and R-squared values are shown in the top left corner. Significance threshold is set to P<0.05.


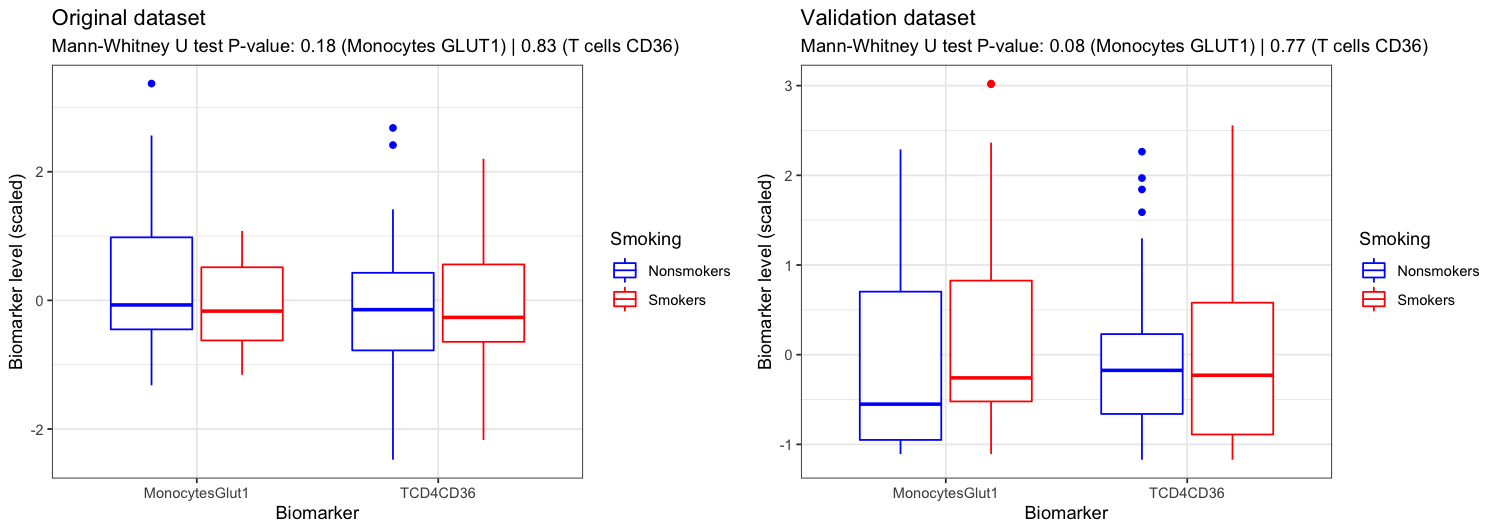


**Supplementary Figure 2. Boxplots of biomarker levels against smoking in the original and validation datasets.** Y-axes show scaled biomarker levels for monocyte glucose transporter 1 (GLUT1) and CD4^+^ T cell fatty acid translocase (CD36). Non-smokers are marked in blue, and smokers are marked in red. Boxplots represent the median, interquartile range, and minimum and maximum values, excluding outliers (dots). Mann-Whitney U test P values used to evaluate differences in biomarker levels between the groups are marked in the top left corner. Significance threshold is set to P<0.05.


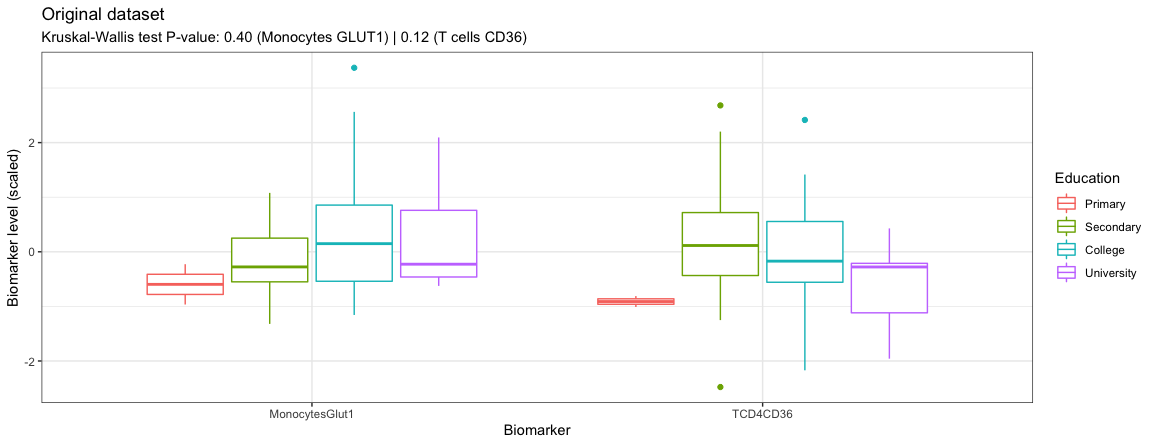


**Supplementary Figure 3. Boxplots of biomarker levels against education in the original dataset.** Y-axis shows scaled biomarker levels for monocyte glucose transporter 1 (GLUT1) and CD4^+^ T cell fatty acid translocase (CD36). Primary education is marked in red, secondary education in green, college level education in blue, and university education in purple. ‘College’ includes the Dutch middelbaar beroepsonderwijs (MBO) and hoger beroepsonderwijs (HBO) education; ‘University’ includes the Dutch wetenschappelijk onderwijs (WO) education. Boxplots represent the median, interquartile range, and minimum and maximum values, excluding outliers (dots). Kruskal-Wallis test P values used to evaluate differences in biomarker levels between the groups are marked in the top left corner. Significance threshold is set to P<0.05.


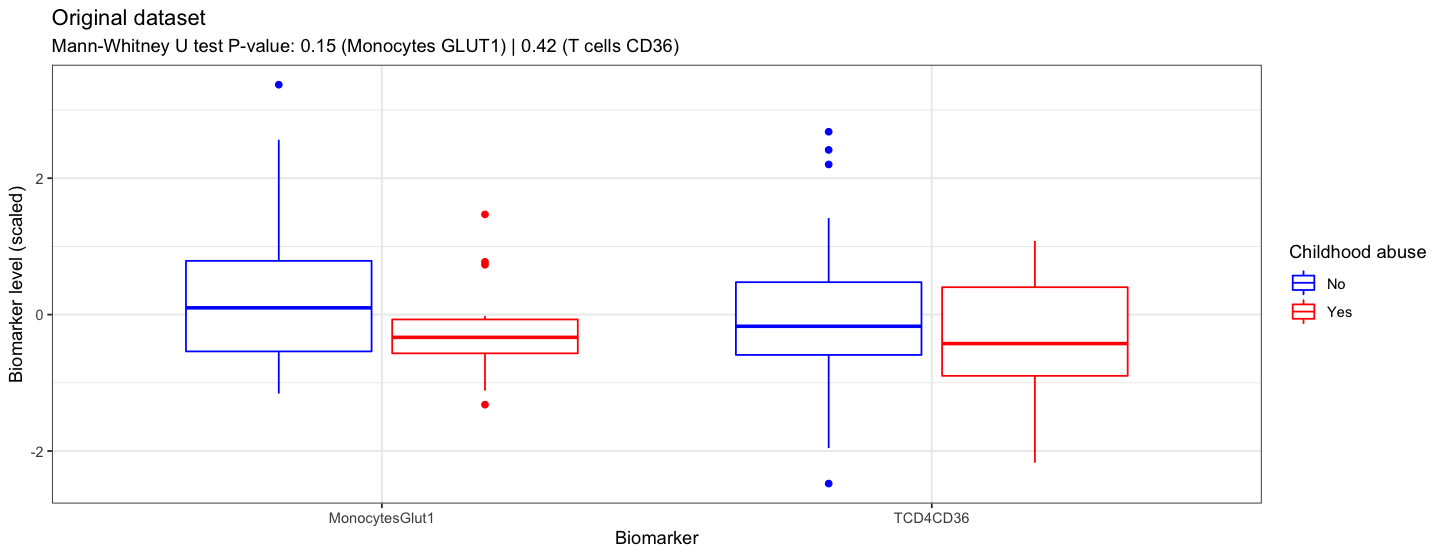


**Supplementary Figure 4. Boxplots of biomarker levels against childhood abuse in the original dataset.** Y-axis shows scaled biomarker levels for monocyte glucose transporter 1 (GLUT1) and CD4^+^ T cell fatty acid translocase (CD36). Biomarker levels of participants with no recorded childhood abuse are marked in blue, and participants with recorded childhood abuse in red. Boxplots represent the median, interquartile range, and minimum and maximum values, excluding outliers (dots). Mann-Whitney U test P values used to evaluate differences in biomarker levels between the groups are marked in the top left corner. Significance threshold is set to P<0.05.


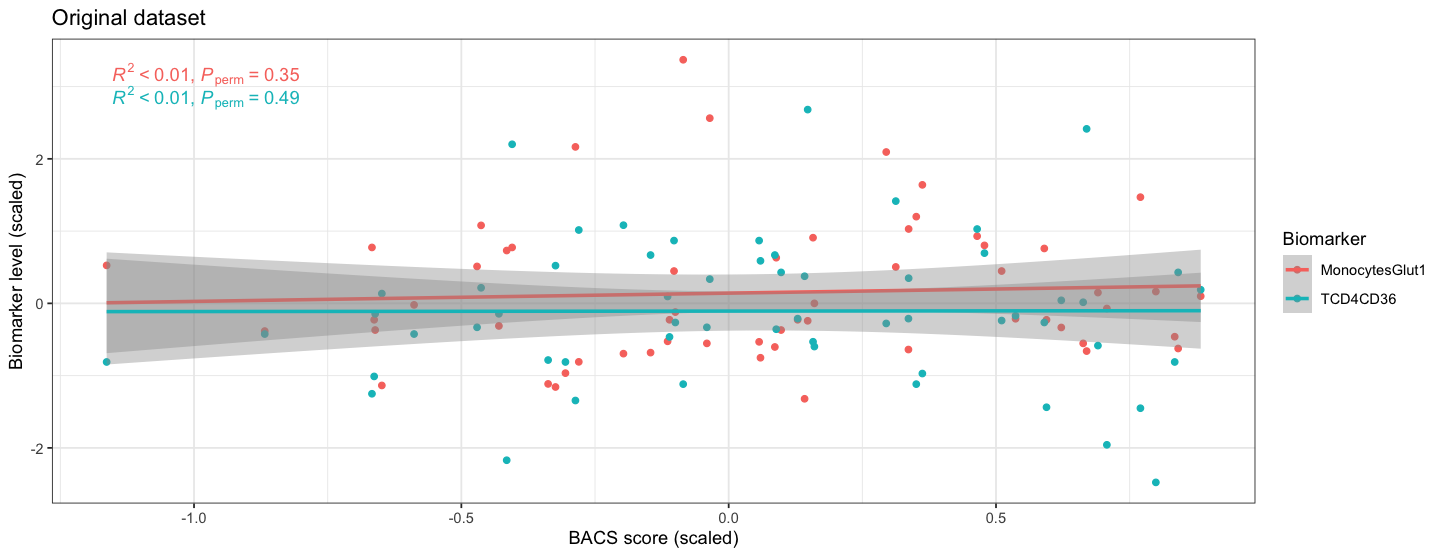


**Supplementary Figure 5. Biomarker levels against cognition scores in the original dataset.** Biomarker levels (Z-scores) are marked on the Y-axis, and cognition score is shown on the X-axis, representing the Brief Assessment of Cognition in Schizophrenia (BACS) total scores. Monocytes glucose transporter 1 (GLUT1) is marked in red and CD4^+^ T cell fatty acid translocase (CD36) is shown in blue. A linear regression line is fit for both biomarkers against both covariates, 95% confidence intervals are represented by the shaded areas, and P values and R-squared values are shown in the top left corner. Significance threshold is set to P<0.05.
